# High Production of LukMF’ in *Staphylococcus aureus* Field Strains Is Associated with Clinical Bovine Mastitis

**DOI:** 10.3390/toxins10050200

**Published:** 2018-05-15

**Authors:** Jurriaan Hoekstra, Victor Rutten, Laura Sommeling, Tine van Werven, Mirlin Spaninks, Birgitta Duim, Lindert Benedictus, Gerrit Koop

**Affiliations:** 1 Department of Infectious Diseases and Immunology, Faculty of Veterinary Medicine, Utrecht University, Yalelaan 1, 3584 CL Utrecht, The Netherlands; v.rutten@uu.nl (V.R.); l.e.i.sommeling@uu.nl (L.S.); B.Duim@uu.nl (B.D.); L.Benedictus1@uu.nl (L.B.); 2 Department of Farm Animal Health, Faculty of Veterinary Medicine, Utrecht University, Yalelaan 7, 3584 CL Utrecht, The Netherlands; T.vanWerven@uu.nl (T.v.W.); M.P.Spaninks@uu.nl (M.S.); G.Koop@uu.nl (G.K.); 3 Department of Veterinary Tropical Diseases, Faculty of Veterinary Science, University of Pretoria, Private Bag X04, Onderstepoort 0110, South Africa; 4 University Farm Animal Practice, 3481 LZ Harmelen, The Netherlands; 5 Division of Infection and Immunity, The Roslin Institute, The University of Edinburgh, Easter Bush, Midlothian EH25 9RG, UK

**Keywords:** *Staphylococcus aureus*, bovine mastitis, clinical severity, LukMF’, repressor of toxins, phage encoded leukocidin

## Abstract

*Staphylococcus aureus*, a major cause of bovine mastitis, produces a wide range of immune-evasion molecules. The bi-component leukocidin LukMF’ is a potent killer of bovine neutrophils in vitro. Since the role of LukMF’ in development of bovine mastitis has not been studied in natural infections, we aimed to clarify whether presence of the *lukM-lukF’* genes and production levels of LukMF’ are associated with clinical severity of the disease. *Staphylococcus aureus* was isolated from mastitis milk samples (38 clinical and 17 subclinical cases) from 33 different farms. The *lukM*-*lukF’* genes were present in 96% of the isolates. Remarkably, 22% of the *lukM-lukF’*-positive *S. aureus* isolates displayed a 10-fold higher in vitro LukMF’ production than the average of the lower-producing ones. These high producing isolates were cultured significantly more frequently from clinical than subclinical mastitis cases. Also, the detection of LukM protein in milk samples was significantly associated with clinical mastitis and high production in vitro. The high producing LukMF’ strains all belonged to the same genetic lineage, *spa*-type t543. Analysis of their global toxin gene regulators revealed a point mutation in the Repressor of toxins (*rot*) gene which results in a non-functional start codon, preventing translation of *rot*. This mutation was only identified in high LukMF’ producing isolates and not in low LukMF’ producing isolates. Since *rot* suppresses the expression of various toxins including leukocidins, this mutation is a possible explanation for increased LukMF’ production. Identification of high LukMF’ producing strains is of clinical relevance and can potentially be used as a prognostic marker for severity of mastitis.

## 1. Introduction

Mastitis—inflammation of the mammary gland—is a major cause of economic losses in the dairy industry associated with costs of treatment, reduced milk yield, discarded milk and premature culling of animals [1,2]. In addition, mastitis has severe impact on animal welfare [3]. *Staphylococcus aureus* is one of the major causes of mastitis in cows [4]. These infections are mostly subclinical and often chronic, but may also result in clinical mastitis [5]. *Staphylococcus aureus* possesses many virulence factors, some of which enable it to manipulate the innate and adaptive immune responses of the host [6]. These virulence factors vary widely between lineages [7] and include immunomodulatory proteins [8], proteases [9], factors that impede phagocytosis [10] and cytotoxins [11]. Some virulence factors are phage-encoded which allows horizontal transfer of virulence genes between bacteria, resulting in genetic diversity between *S. aureus* lineages [12]. An important group of *S. aureus* immune evasion molecules is that of the leukocidins: pore-forming, bi-component toxins that specifically target immune cells [13]. So far, seven different leukocidins have been described [13,14], of these, the phage-encoded leukocidins Panton-Valentine leukocidin (PVL), LukPQ, and LukMF’ most strongly affect immune cells from a limited host species range [6,13,14]. LukMF’ is almost exclusively present on prophages carried by *S. aureus* strains of ruminant origin [15,16,17] and it is a potent killer of bovine neutrophils, macrophages and monocytes, but not of human neutrophils [18,19]. LukM binds to the CCR1 receptor, which is highly present on bovine neutrophils, but absent on human neutrophils [19]. Since neutrophils are key players in initial immune responsiveness during inflammation of the mammary gland [20], it is expected that potent killing of neutrophils by LukMF’ reduces its effectiveness and, therefore, influences the clinical outcome of infection. Indeed, experimental intramammary challenge with high LukMF’ producing *S. aureus* strains resulted in more severe mastitis compared to challenge with intermediate producing *S. aureus* strains [21]. Also, high LukM levels in milk have been associated with clinical mastitis [21]. It is, however, unclear whether differences in LukMF’ production are of clinical significance in natural infections.

In this study, we investigated the impact of LukMF’ on clinical severity of bovine mastitis under field conditions. We identified a genetic lineage of *S. aureus* with increased LukMF’ production that is associated with clinical rather than subclinical mastitis. In this lineage, we found a nonsense mutation in the start codon of the global expression regulator Repressor of toxins (*rot*)*.* This mutation is the likely cause of increased toxin production associated with severity of clinical signs of mastitis.

## 2. Results

### 2.1. Prevalence of lukM-lukF’ Genes among Bovine Mastitis Isolates

Fifty-five *S. aureus* isolates were collected at 33 Dutch dairy farms by bacteriological culture of milk samples from clinical (*n* = 37) and subclinical (*n* = 18) cases of bovine mastitis and the presence of the *femA*, *lukM*, and *lukF’* genes was detected using PCR. All isolates were positive for the *S. aureus*-specific gene *femA*, confirming that the selected bacteria were *S. aureus*, and 53/55 (96%) of isolates were positive for *lukM* and *lukF’*. The proportion of *lukM-lukF’* positive *S. aureus* was similar for clinical (37/38, 97%) and subclinical (16/17, 94%) mastitis cases.

### 2.2. Production of LukM In Vitro and In Vivo

**Figure 1:**
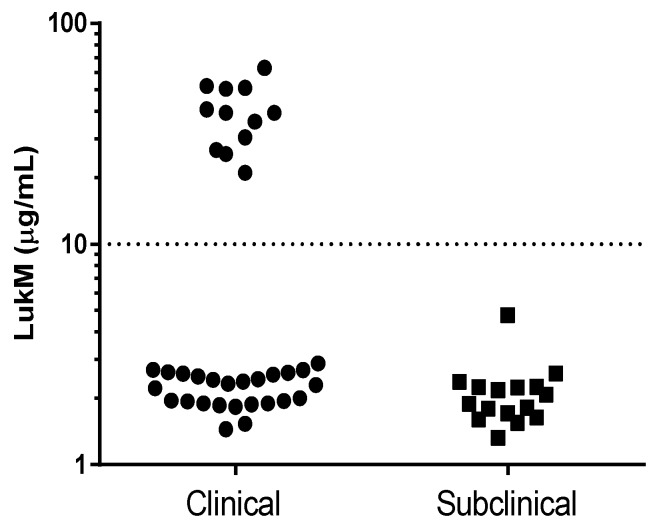
LukM levels in vitro after eight hours of culture of 53 *lukM-lukF’* positive *S. aureus* isolates from milk of cows with clinical or subclinical mastitis, measured by ELISA.

The in vitro LukMF’ production potential was investigated by growing *S. aureus* isolates for eight hours under controlled conditions and measuring the LukM concentration in supernatant using ELISA. All *lukM-lukF’* positive *S. aureus* isolates produced LukM in vitro, with levels ranging from 1.5 to 60 µg/mL. The production levels had a clear bimodal distribution (Figure 1). Based on this distribution, isolates were categorized into two groups: LukMF’ high producers (>10 µg/mL) and LukMF’ low producers (<10 µg/mL). Most *S. aureus* (*n* = 41) were LukMF’ low producers (mean = 2.2 µg/mL LukM, SD = 0.6). The remaining isolates (*n* = 12) were LukMF’ high producers (mean = 39.6 µg/mL LukM, SD = 12.6) and were significantly more often isolated from cases of clinical mastitis (Fisher’s exact test, *p* = 0.011). Although some of the 55 *S. aureus* isolates originated from the same farm, the 12 high producing isolates were cultured from cows on 12 different farms. To compensate for a possible farm effect, we randomly selected one isolate per farm (*n* = 33) and found the same association between the LukMF’ high producing isolates and clinical mastitis (Fisher’s exact test, *p* = 0.033).

**Figure 2:**
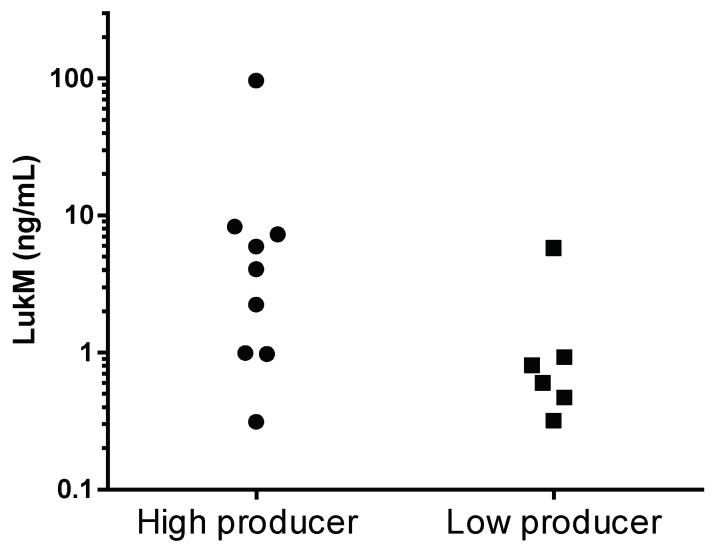
LukM concentration in milk samples of mastitis cases (in vivo) caused by LukMF’ high (>10 µg/mL production in vitro after eight hours of culture) and low (<10 µg/mL production in vitro after eight hours of culture) producing *S. aureus* (*p* = 0.049, Mann–Whitney test).

Next, in vivo LukMF’ production during mastitis was assessed by measuring LukM in the milk samples corresponding to each of the *S. aureus* isolates. Eight milk samples (seven clinical and one subclinical) could not be tested for LukM because the milk was clotted and therefore not fit for use in the ELISA, or because insufficient milk was available. LukM was detected in 15/45 milk samples, with concentrations ranging from 0.31 ng/mL to 96 ng/mL, significantly more often in milk from clinical mastitis cases (13/29 = 45%) than from subclinical cases (2/16 = 12%, Fisher’s exact test, *p* = 0.046) and in samples that yielded LukMF’ high producing isolates (9/11 = 82%) compared to samples that yielded LukMF’ low producing isolates (6/34 = 18%, Fisher’s exact test, *p* < 0.001). After again selecting one isolate per farm, the association between high in vitro production and LukM in milk, ex vivo, remained (Fisher’s exact test, *p* = 0.007), but the association between LukM in milk and clinical severity was no longer statistically significant (Fisher’s exact test, *p* = 0.22). The average LukM concentration in milk of cows that hosted LukMF’ high producing isolates (mean = 14.2 ng/mL LukM, SD = 31.1) was higher than of those carrying LukMF’ low producing isolates (mean = 1.49 ng/mL LukM, SD = 2.12) (Mann–Whitney test, *p* = 0.049) (Figure 2).

### 2.3. Genotyping of S. aureus Isolates

All isolates were *spa*-typed to investigate whether differences in LukMF’ production associated with certain genetic lineages. Table 1 gives the *spa*-types identified in our isolate collection, and shows that all high producing *S. aureus* isolates belonged to *spa*-type t543. Whole genome sequencing was performed on a subset of seven isolates, selected based on differences in LukM production levels, *spa*-type and farm. Sequencing results showed that all three *spa*-type t543 strains belonged to multilocus sequence type (ST) 479, which belongs to clonal complex (CC) 479. The three *spa*-type t529 isolates all belonged to CC151, with two isolates identified as ST151 and one as ST504, a double-locus variant (DLV) of ST151. The single *spa*-type t1403 strain belonged to ST133 (CC133).

### 2.4. Analysis of the lukM-lukF’ Operon and saeS, saeR, rot Genes

To identify genetic factors that were associated with the LukMF’ production phenotype, we compared whole genome sequences of three high producing and four low producing *S. aureus* isolates. First, the prophages containing *lukM-lukF*’ were identified using PHAST [22]. Prophages encoding *lukM-lukF*’ were indeed present in all sequenced isolates, and were identified by PHAST to be most similar to reference phage phiPV83 (Genbank accession NC_002486.1). Prophages from CC479 and CC133 isolates were very similar to each other in size and gene content, but both differed from the smaller CC151 prophage. Variations were observed in *lukM* (four synonymous and five non-synonymous single nucleotide polymorphisms (SNPs)) and *lukF*’ (three synonymous and six non-synonymous SNPs) among the different CCs (Supplementary Figure S1a,b). The putative promotor region of the *lukM-lukF’* operon (up to 200 bp upstream from the start codon) contained five SNPs, but none were exclusively present in high producing CC479 strains (Supplementary Figure S1c). 

Next, we examined genes putatively involved in the regulation of *lukM* expression. Genes involved in LukMF’ expression are unknown, but analogous to the regulation of the expression of PVL, we investigated Repressor of toxins (*rot*) and the exoprotein two-component system SaeRS (*saeS* and *saeR*) [23,24]. These genes were present in all sequenced strains and no variation was found in the *saeR* gene, whereas a single non-synonymous SNP (not associated with CC479) was found in the *saeS* gene (data not shown). Two non-synonymous SNPs (position 2 and 452) were found in the *rot* gene, exclusively present in the CC479 isolates. The SNP at position 2 renders the *rot* gene non-functional due to the loss of a start codon. To corroborate these findings, the region surrounding the start codon of *rot* was identified in five additional *S. aureus* isolates (three LukMF’ high producing isolates and two LukMF’ low producing isolates) using PCR and sequencing. In these additional isolates, the mutation in the *rot* start codon was also only present in the high producing *S. aureus* isolates (Supplementary Figure S1d).

## 3. Discussion

We observed a high *lukM-lukF*’ carriage (96%) among *S. aureus* field isolates cultured both from cases of clinical and subclinical mastitis. A subpopulation of *S. aureus*, *spa*-type t543-ST479, with a very high in vitro LukMF’ production was significantly associated with clinical rather than subclinical mastitis. A mutation in the *rot* gene, leading to loss of the primary start codon, found exclusively in *spa*-type t543-ST479 isolates, may be functionally linked to the increased LukMF’ production. 

All sequence types and *spa*-types identified in this study have previously been associated with bovine mastitis [25,26,27,28,29]. Several authors have reported about sequence types and *lukM-lukF*’ carriage of *S. aureus*, and this reveals that it is strongly associated with specific ruminant-associated clonal complexes, namely CC151, CC133, CC705, and CC479 [16,30,31,32,33,34] (see supplementary Table S1 for a detailed overview). Within the bovine associated CC97, only one specific lineage (ST352-CC97) showed a high prevalence of *lukM-lukF*’ [30,31]. This demonstrates that the carriage of the *lukM-lukF*’ harboring (pro)phage [17] is lineage specific, since the genes are only present in certain sequence types and absent in others within the same CC. 

LukMF’ is a potent killer of bovine neutrophils, which play a critical role in the innate immune defense against *S. aureus* [5,19]. We hypothesize that killing of neutrophils by LukMF’ reduces the overall phagocytic activity in the mammary gland, resulting in survival of *S. aureus*, and hence pro-inflammatory responsiveness increasing the clinical severity of mastitis. In our study, LukMF’ high production in vitro by ST479 *S. aureus* is indeed associated with clinical rather than subclinical mastitis. High production was also strongly correlated with substantial levels of LukM present in milk in vivo. Still, also the presence of LukMF’ low producing *S. aureus* in milk may lead to detectable levels. Although differences in bacterial load of LukMF’ producing bacteria in the mammary gland could also explain differences in milk LukM levels, we assume that ST479 are also LukMF’ high producers in vivo during the course of infection. In a recent study, cattle were challenged intramammary with LukMF’ high and low producing *S. aureus* strains [21]. Quarters challenged with the high producer developed more severe clinical symptoms and higher bacterial loads compared to the other, low producing, strains [21].

Although, to our knowledge, the regulation of LukMF’ expression has not been studied, it is plausible to assume that the regulation system of LukMF’ expression is similar to that of other leukocidins, which are controlled by global gene regulators Agr, Rot and SaeRS [13]. We observed a mutation in the *rot* start codon that was strongly associated with high LukMF’ production. Rot is a global regulator of *S. aureus* virulence gene expression and can directly bind to the promotor region of various toxin genes, such as *hla*, *hlgC-hlgB*, *lukE-lukD*, and *lukA-lukB* [23,35]. Mutations in *rot* can both activate or repress gene expression, depending on the site of the mutation and the target gene [35]. The expression of leukocidins (LukAB, LukED, PVL) by *S. aureus* increases when the *rot* gene is made inoperative [23,36]. The hypervirulence of the community-associated methicillin-resistant *S. aureus* (CA-MRSA) clone USA500 is believed to be a result of increased leukocidin (LukAB, LukED, hlgCB) production compared to other CA-MRSA, induced by an insertion in the promotor region of *rot* that prevents expression of this gene [36]. The LukMF’ high producing strain used in a previous study [21] also contained the same mutation in *rot* as the one identified in our work. This demonstrates that the absence of, or impaired or altered function of *rot* likely explains the increased LukMF’ production of ST479 strains observed in our study. To further substantiate this, ST479 could be complemented with a functional *rot* copy which should lead to decreased LukMF’ production compared to the WT ST479. Likewise, *rot* knockout strains of ST151 or ST133 are expected to produce higher amounts of LukMF’ compared to the WT. These strains could also be used to identify how this mutation in rot affects the regulation of other leukocidins. Because of the limited geographic range from which our samples originated, it is unclear how prevalent the mutation in *rot* is in CC479 isolates cultured from cattle in other countries. Still, the strain S1444 [21], a high producing CC479 isolate was originally cultured from a German sample, suggesting that the mutation is not restricted to the region of this study.

Mastitis milk samples used in our study as source of *S. aureus* isolates were submitted by farmers, sometimes in consultation with their veterinarian, hence not randomly collected and not likely to be representative for the population in the field. Due to that approach, under- or overestimation of the actual proportions of LukMF’ high producing isolates in the population as well as of isolates belonging to the various *spa*-types cannot be excluded. The association between high LukMF’ production and clinical versus subclinical mastitisis, however, not likely to be affected by this sampling bias, but the clinical importance of this association in terms of the population attributable fraction depends on the prevalence of LukMF’ high producing isolates in the field and cannot be calculated from our data.

Since the *rot* gene is part of the core genome of *S. aureus*, transfer of the ability of ST479 to produce high levels of LukMF’ seems unlikely. It is unclear if increased production of LukMF’ by ST479 also increases the transmission of this lineage in the population, as a severe clinical infection is expected to result in a quicker death or culling from the herd of the host or quicker treatment with antibiotics, reducing the chances to further spread in the population. In previous studies, ST479 made up 26% of Dutch [28] and 17% of German *S. aureus* mastitis isolates [16], suggesting that this lineage can persist within a population despite its association with clinical mastitis.

Screening tools, like loop-mediated isothermal amplification (LAMP) [37], currently exist that allow for quick identification of mastitis pathogens [38]. However, these tools mostly identify the pathogen on a species level. Since our research shows that the sequence type of *S. aureus* is strongly associated to the type of mastitis, sequence typing of mastitis isolates would enable farmers to implement specific interventions in case of infection with the high LukMF’ producing lineages.

## 4. Materials and Methods

### 4.1. Collection of S. aureus Bovine Mastitis Isolates

Quarter milk samples from cows with subclinical or clinical mastitis were aseptically collected by farmers belonging to the University Farm Animal Practice (Harmelen, the Netherlands) and sent in for bacteriological culture and species identification, which were performed according to National Mastitis Council protocols [39]. As participating farms (*n* = 33) belonged to the same veterinary practice, the farms were geographically clustered around the Utrecht region in the Netherlands. Samples were collected between April 2014 and December 2015. Clinical or subclinical mastitis was diagnosed by the farmer, with clinical mastitis defined as visibly abnormal appearance of the udder, the milk or both. Subclinical mastitis was characterized by absence of clinical signs and generally were animals with a high somatic cell count. A total of 55 *S. aureus* positive milk samples from cases of bovine mastitis (38 clinical and 17 subclinical cases) were used for this study and stored at −18 °C before further use. After thawing of the milk samples, 200 µL aliquots of the *S. aureus* positive milk samples were plated on sheep blood agar plates and cultured overnight at 37 °C to isolate fresh bacterial colonies. Of milk samples showing signs of a clinical mastitis (clots, flakes, discolored milk), a smaller volume of 50 µL was used to prevent bacterial overgrowth of the plate. Single colonies were picked and added to 2 mL T1438 Todd Hewitt Broth (THB) (Sigma, St. Louis, MO, USA) and incubated overnight at 37 °C with agitation. Bacterial glycerol stocks (25% glycerol) were made by adding 0.5 mL of bacterial broth to 0.5 mL 50% glycerol solution in distilled water. Stocks were stored at −80 °C before use in further experiments.

### 4.2. DNA Extraction and Amplification of lukM, lukF’, femA and rot Genes

Bacterial isolates were plated from glycerol stocks on blood agar plates and cultured overnight at 37 °C. Single colonies were picked for DNA extraction and washed in 1 mL distilled water. After centrifugation (17,000× *g* for 1 min), bacteria were resuspended in 200 µL distilled water and heated at 100 °C for 10 min, centrifuged at 17,000× *g* for 1 min, and diluted 1:10 in distilled water and stored at −20 °C.

Primers to amplify *femA*, *lukM*, *lukF*’ and *rot* were designed or taken from literature (Table 2). The primers for *femA* and *lukF*’ were used together in a duplex PCR, and the other primers (*rot*, *lukM*) in separate, single PCR. The reaction was performed in a total volume of 25 µL containing 10 µL 1:10 diluted boiled DNA sample, 5 µL GoTaQ Green buffer 5× (Promega, Madison, WI, USA), 1.5 mM MgCl_2_ (Promega), 0.2 mM dNTPs (Promega, Madison, WI, USA), 0.4 µM of the *lukF*’ primer pair and 0.6 µM of the other primer pairs (Invitrogen, Carlsbad, CA, USA) and 0.625 U of GoTaQ DNA polymerase (Promega, Madison, WI, USA). After an initial denaturation step at 95 °C for 2 min, 35 cycles (30 s at 95 °C, 35 s at various annealing temperatures (Table 2, column 4) and 35–60 s at 72 °C) were performed in a T100 Thermal Cycler (Bio-Rad, Hercules, CA, USA). Electrophoresis on 1.5% agarose gel was used to visualize PCR products.

### 4.3. In Vitro and In Vivo LukM Production

Bacteria were cultured from glycerol stock on blood agar plates overnight at 37 °C. Single colonies were picked and added to 1.5 mL THB. Bacteria were incubated with agitation for 30 min at 37 °C. Optical density (OD) at 600 nm was measured and samples were diluted to an OD of 0.01 in 2.5 mL of THB. Next, bacteria were incubated with agitation for 8 h at 37 °C. After incubation, samples were centrifuged (4000× *g* for 10 min) and supernatant was collected. The supernatant was sterilized using a microfilter (0.20 µm; Corning Incorporated, Corning, NY, USA) and stored at −20 °C before use in further experiments. Bacterial supernatants were produced in triplicate using separate, single colonies from the same cultured plate.

LukM in supernatant and bovine mastitis milk samples was measured by ELISA, according to the method described by Vrieling et al. [21]. In short, LukM is captured using LukM specific polyclonal bovine IgG isolated from the colostrum of a cow with high LukM antibody titers, and captured LukM is detected using the LukM specific monoclonal antibody LM43.F8 [21]. Milk samples were heated to 95 °C for 10 min to prevent interference from antibodies in the milk.

### 4.4. Genotyping of Mastitis Strains and Genomic Analyses

The polymorphic X-region of the Staphylococcal Protein A (*spa*) gene of all *S. aureus* isolates was amplified according to the Ridom StaphType standard protocol (www.ridom.org). PCR amplicons were purified using ExoSAP-IT PCR Cleanup Reagent (Affymetrix, Santa Clara, CA, USA) according to manufacturer’s instructions and sequenced using Sanger sequencing (Baseclear, Leiden, The Netherlands). BioNumerics v7.5 software (Applied Maths, Sint-Martens-Latem, Belgium) was used to analyze sequence data and to assign *spa*-types.

Whole genome sequencing was performed on seven isolates, selected from LukMF’ high and low producers of different *spa*-type, and each selected isolate originated from a different farm. DNA was isolated with the Ultra Clean Microbial DNA isolation kit (Mo-Bio, Carlsbad, CA, USA). MiSeq sequencing (Illumina, San Diego, CA, USA) was performed at the Utrecht Sequencing Facility (UMC Utrecht, the Hubrecht institute and Utrecht University, the Netherlands), using 300 bp paired end reads. Reads were assembled into a scaffold genome using SPAdes v3.1.1. [41]. This Whole Genome Shotgun project has been deposited at DDBJ/ENA/GenBank under the accession QFCT00000000-QFCZ00000000. The version described in this paper is the first version. MLST was determined using the MLST tool of the Center of Genomic Epidemiology (accessible on https://cge.cbs.dtu.dk/services/MLST/) [42]. Prophages containing the *LukM-lukF*’ genes were identified using PHAST (accessible on http://phast.wishartlab.com/index.html) [22]. The *lukM-lukF*’ encoding gene sequences and putative promotor region (200 bp upstream of start *LukM* gene) were identified by BLASTN using reference sequences for *lukM *(GenBank accession: 1262967) and *lukF*’ (GenBank accession: 1262954). Sequences for candidate LukMF’ regulator genes were extracted from available genomes using reference gene sequences of *rot* (Genbank accession: AF189239.2) and *saeRS* locus (Genbank accession: AF129010.1). Nucleic acid sequences were translated to their corresponding protein sequences using EMBOSS Transeg software (accessible on http://www.ebi.ac.uk/Tools/st/emboss_transeq/). Gene and protein sequences were aligned using MegALIGN Pro software (DNAstar Incorporate, Madison, WI, USA).

### 4.5. Statistical Analysis

Statistical analysis was performed using GraphPad Prism 7 software (GraphPad Software, La Jolla, CA, USA). In vitro differences in LukM levels between clinical/subclinical isolates and in vivo LukM levels between LukMF’ high/low producers were compared using the Mann–Whitney U test. The associations between in vitro LukMF’ production levels, presence of detectable LukM in milk and mastitis type were tested using Fisher’s exact test. A subset of the dataset with a single sample per farm was assembled using the random number generator function in Microsoft Excel (Microsoft Corporation, Redmond, WA, USA).

## Figures and Tables

**Table 1:** *Spa*-types and in vitro LukMF’ production of field isolates from bovine clinical and subclinical mastitis cases.

*Spa*-Type	Isolates N	Clinical Mastitis N (%)	*lukM-lukF’* Postive Isolates N (%) ^1^	High LukMF’ Production Isolates N (%) ^2^	Clonal Complex ^3^
t529	40	23 (58)	40 (100)	0 (0)	CC151
t543	12	12 (100)	12 (100)	12 (100)	CC479
t524	1	1 (100)	0 (0)	0 (0)	ND ^4^
t1403	1	1 (100)	1 (100)	0 (0)	CC133
t015	1	0 (0)	0 (0)	0 (0)	ND ^4^
**Total**	**55**	**37 (67)**	**53 (96)**	**12 (22)**	

^1^ Number of *lukM*-*lukF*’ positive isolates. Genes detected by PCR. ^2^ Number of samples with >10 µg/mL LukMF’ production after eight hours culture, measured in supernatant by ELISA. ^3^ Clonal complex determined by MLST based on whole genome sequences of subset of isolates from this *spa*-type. ^4^ Not determined.

**Table 2:** Primers and annealing temperature used in this study.

Gene	Sequence	Product Size (bp)	Annealing Temperature (°C)	Reference
*femA*	f: 5′-tgcctttacagatagcatgcca-3′	142	59.5	[40]
	r: 5′-agtaagtaagcaagctgcaatgacc-3′			
*lukM*	f: 5′-aaacgcgcagttaataaaaag-3′	975	55	This study
	r: 5′-agcattaggtcctcttgtcg-3′			
*lukF’*	f: 5′-actcaggctatacccaaccca-3′	472	59.5	This study
	r: 5′-cgagctactctgtctgccac-3′			
*rot*	f: 5′-accaatttagcctcattcggtttg-3′	705	55	This study
	r: 5′-catcgtcaacaggacgctct-3′			

## Supplementary Materials

The following are available online at http://www.mdpi.com/2072-6651/10/5/200/s1, Figure S1: (a) Alignment of *lukM* of seven *S. aureus* isolates obtained from cases of bovine mastitis; (b) Alignment of *lukF’* of seven *S. aureus* isolates obtained from cases of bovine mastitis; (c) Alignment of the putative promotor region of *lukM-lukF’* operon of seven *S. aureus* isolates obtained from cases of bovine mastitis; (d) Alignment of the first 20 nucleotides of *rot* in 12 *S. aureus* isolates obtained from cases of bovine mastitis. Table S1: Number of *S. aureus* of different lineages found among bovine isolates, with percentage of *lukM-lukF*’ positive *S. aureus* among these lineages.
