# Supplementary material for: High Production of LukMF’ in Staphylococcus aureus Field Strains Is Associated with Clinical Bovine Mastitis
Source: Toxins (Basel). 2018 May 15;10(5):200. doi: 10.3390/toxins10050200 (PMC5983256; doi:10.3390/toxins10050200)
Supplement: Supplementary file 1 [file toxins-10-00200-s001.pdf]

Supplementary Materials: High Production of LukMF′ in Staphylococcus aureus Field Strains Is Associated with Clinical Bovine Mastitis

Jurriaan Hoekstra, Victor Rutten, Laura Sommeling, Tine van Werven, Mirlin Spaninks, Birgitta Duim, Lindert Benedictus, Gerrit Koop

```
CC133-s139    1 ATGTTTAAGAGAAAATTATTAGTTACAACTTTGTCGCTAGGTCTAATTGTCCCTATAGCTACACCATTTCAAGGCTCTAAGGCTACTACT
CC151-s31     1 ATGTTTAAGAGAAAATTATTAGTTACAACTTTGTCGCTAGGTCTAATTGTTCCTATAGCTACACCATTTCAAGGTTCTAAGGCTACTACT
CC151-s483    1 ATGTTTAAGAGAAAATTATTAGTTACAACTTTGTCGCTAGGTCTAATTGTTCCTATAGCTACACCATTTCAAGGTTCTAAGGCTACTACT
CC151-s909    1 ATGTTTAAGAGAAAATTATTAGTTACAACTTTGTCGCTAGGTCTAATTGTTCCTATAGCTACACCATTTCAAGGTTCTAAGGCTACTACT
CC479-s15     1 ATGTTTAAGAGAAAATTATTAGTTACAACTTTGTCGCTAGGTCTAATTGTCCCTATAGCTACACCATTTCAAGGCTCTAAGGCTACTACT
CC479-s28     1 ATGTTTAAGAGAAAATTATTAGTTACAACTTTGTCGCTAGGTCTAATTGTCCCTATAGCTACACCATTTCAAGGCTCTAAGGCTACTACT
CC479-s58     1 ATGTTTAAGAGAAAATTATTAGTTACAACTTTGTCGCTAGGTCTAATTGTCCCTATAGCTACACCATTTCAAGGCTCTAAGGCTACTACT

CC133-s139   91 AATGCAGAAGATATTGGCGACGATGCAGAAGTGATTAAACGTACGGAAGATGTAAGTAGTAGGAAATGGGGTGTAACACAAAATGTCCAA
CC151-s31    91 AATGCAGAAGATATTGGCGACGATGCAGAAGTGATTAAACGTACGGAAGATGTAAGTAGTAGGAAATGGGGTGTAACACAAAATGTCCAA
CC151-s483   91 AATGCAGAAGATATTGGCGACGATGCAGAAGTGATTAAACGTACGGAAGATGTAAGTAGTAGGAAATGGGGTGTAACACAAAATGTCCAA
CC151-s909   91 AATGCAGAAGATATTGGCGACGATGCAGAAGTGATTAAACGTACGGAAGATGTAAGTAGTAGGAAATGGGGTGTAACACAAAATGTCCAA
CC479-s15    91 AATGCAGAAGATATTGGTGACGATGCAGAAGTGATTAAACGTACGGAAGATGTAAGTAGTAGGAAATGGGGTGTAACACAAAATGTCCAA
CC479-s28    91 AATGCAGAAGATATTGGTGACGATGCAGAAGTGATTAAACGTACGGAAGATGTAAGTAGTAGGAAATGGGGTGTAACACAAAATGTCCAA
CC479-s58    91 AATGCAGAAGATATTGGTGACGATGCAGAAGTGATTAAACGTACGGAAGATGTAAGTAGTAGGAAATGGGGTGTAACACAAAATGTCCAA

CC133-s139  181 TTTGATTTCGTAAAAGATAAAAAATATAACAAAGACGCATTAATTATTAAGATGCAAGGTTTTATCAATTCTAGGACAACTTTCAATGAT
CC151-s31   181 TTTGATTTCGTAAAAGATAAAAAATATAACAAAGACGCATTAATTATTAAGATGCAAGGTTTTATCAATTCTAGGACAACTTTCAATGAT
CC151-s483  181 TTTGATTTCGTAAAAGATAAAAAATATAACAAAGACGCATTAATTATTAAGATGCAAGGTTTTATCAATTCTAGGACAACTTTCAATGAT
CC151-s909  181 TTTGATTTCGTAAAAGATAAAAAATATAACAAAGACGCATTAATTATTAAGATGCAAGGTTTTATCAATTCTAGGACAACTTTCAATGAT
CC479-s15   181 TTTGATTTCGTAAAAGATAAAAAATATAACAAAGACGCATTAATTATTAAGATGCAAGGTTTTATCAATTCTAGGACAACTTTCAATGAT
CC479-s28   181 TTTGATTTCGTAAAAGATAAAAAATATAACAAAGACGCATTAATTATTAAGATGCAAGGTTTTATCAATTCTAGGACAACTTTCAATGAT
CC479-s58   181 TTTGATTTCGTAAAAGATAAAAAATATAACAAAGACGCATTAATTATTAAGATGCAAGGTTTTATCAATTCTAGGACAACTTTCAATGAT

CC133-s139  271 GTTAAACAAAATAGAGCAAATAAAAGAATGGTTTGGCCATTTCAATATAATATCGGTCTTACATCAAAAGACGAAAATACGAGCTTAATC
CC151-s31   271 GTTAAACAAAATAGAGCAAATAAAAGAATGGTTTGGCCATTTCAATATAATATCGGTCTTACATCAAAAGACCAAAATACGAGCTTAATC
CC151-s483  271 GTTAAACAAAATAGAGCAAATAAAAGAATGGTTTGGCCATTTCAATATAATATCGGTCTTACATCAAAAGACCAAAATACGAGCTTAATC
CC151-s909  271 GTTAAACAAAATAGAGCAAATAAAAGAATGGTTTGGCCATTTCAATATAATATCGGTCTTACATCAAAAGACCAAAATACGAGCTTAATC
CC479-s15   271 GTTAAACAAAATAGAGCAAATAAAAGAATGGTTTGGCCATTTCAATATAATATCGGTCTTACATCAAAAGACCAAAATACGAGCTTAATC
CC479-s28   271 GTTAAACAAAATAGAGCAAATAAAAGAATGGTTTGGCCATTTCAATATAATATCGGTCTTACATCAAAAGACCAAAATACGAGCTTAATC
CC479-s58   271 GTTAAACAAAATAGAGCAAATAAAAGAATGGTTTGGCCATTTCAATATAATATCGGTCTTACATCAAAAGACCAAAATACGAGCTTAATC

CC133-s139  361 AATTATCTTCCTAAAAATAAAATAGAAACAGTTGATGTTGGTCAAACTTTAGGATATAACATTGGAGGTAAATTCCAGTCAGTACCATCT
CC151-s31   361 AATTATCTTCCTAAAAATAAAATAGAAACAGTTGATGTTGGTCAAACTTTAGGATATAACATTGGAGGTAAATTCCAGTCAGCACCATCT
CC151-s483  361 AATTATCTTCCTAAAAATAAAATAGAAACAGTTGATGTTGGTCAAACTTTAGGATATAACATTGGAGGTAAATTCCAGTCAGCACCATCT
CC151-s909  361 AATTATCTTCCTAAAAATAAAATAGAAACAGTTGATGTTGGTCAAACTTTAGGATATAACATTGGAGGTAAATTCCAGTCAGCACCATCT
CC479-s15   361 AATTATCTTCCTAAAAATAAAATAGAAACAGTTGATGTTGGTCAAACTTTAGGATATAACATTGGAGGTAAATTCCAGTCAGCACCATCT
CC479-s28   361 AATTATCTTCCTAAAAATAAAATAGAAACAGTTGATGTTGGTCAAACTTTAGGATATAACATTGGAGGTAAATTCCAGTCAGCACCATCT
CC479-s58   361 AATTATCTTCCTAAAAATAAAATAGAAACAGTTGATGTTGGTCAAACTTTAGGATATAACATTGGAGGTAAATTCCAGTCAGCACCATCT

CC133-s139  451 ATAGGCGGAAATGGATCATTTAATTATTCTAAGAGTATTAAATATTCCCAAAAGAGTTATGTCAGCGAAGTTGAACAACAAAGCTCAAAA
CC151-s31   451 ATAGGCGGAAATGGATCATTTAATTATTCTAAGAGTATTAAATATTCCCAAAAGAGTTATGTCAGCGAAGTTGAACAACAAAGCTCAAAA
CC151-s483  451 ATAGGCGGAAATGGATCATTTAATTATTCTAAGAGTATTAAATATTCCCAAAAGAGTTATGTCAGCGAAGTTGAACAACAAAGCTCAAAA
CC151-s909  451 ATAGGCGGAAATGGATCATTTAATTATTCTAAGAGTATTAAATATTCCCAAAAGAGTTATGTCAGCGAAGTTGAACAACAAAGCTCAAAA
CC479-s15   451 ATAGGCGGAAATGGATCATTTAATTATTCTAAGAGTATTAAATATTCCCAAAAGAGTTATGTCAGCGAAGTTGAACAACAAAGCTCAAAA
CC479-s28   451 ATAGGCGGAAATGGATCATTTAATTATTCTAAGAGTATTAAATATTCCCAAAAGAGTTATGTCAGCGAAGTTGAACAACAAAGCTCAAAA
CC479-s58   451 ATAGGCGGAAATGGATCATTTAATTATTCTAAGAGTATTAAATATTCCCAAAAGAGTTATGTCAGCGAAGTTGAACAACAAAGCTCAAAA

CC133-s139  541 ACTATTAAGTGGGGGGTTAAAGCAAATTCTTTTGTTATAGCAGGGCATCGATGGTCTGCTTACGATGAATTATTGTTTATAAGAAATACG
CC151-s31   541 ACTATTAAGTGGGGGGTTAAAGCAAATTCTTTTGTTATAGCAGGGCATCGATGGTCTGCTTACGATGAATTATTGTTTATAAGAAATACG
CC151-s483  541 ACTATTAAGTGGGGGGTTAAAGCAAATTCTTTTGTTATAGCAGGGCATCGATGGTCTGCTTACGATGAATTATTGTTTATAAGAAATACG
CC151-s909  541 ACTATTAAGTGGGGGGTTAAAGCAAATTCTTTTGTTATAGCAGGGCATCGATGGTCTGCTTACGATGAATTATTGTTTATAAGAAATACG
CC479-s15   541 ACTATTAAGTGGGGGGTTAGAGCAAATTCTTTTGTTATAGCAGGGCATCGATGGTCTGCTTACGATGAATTATTGTTTATAAGAAATACG
CC479-s28   541 ACTATTAAGTGGGGGGTTAGAGCAAATTCTTTTGTTATAGCAGGGCATCGATGGTCTGCTTACGATGAATTATTGTTTATAAGAAATACG
CC479-s58   541 ACTATTAAGTGGGGGGTTAGAGCAAATTCTTTTGTTATAGCAGGGCATCGATGGTCTGCTTACGATGAATTATTGTTTATAAGAAATACG

CC133-s139  631 ACAAGAGGACCTAATGCTAGAGACTATTTTGTAGACGATAATGAATTGCCCCCTTTAATAACAAGTGGATTTAATCCGTCTTTTATCGCG
CC151-s31   631 ACAAGAGGACCTAATGCTAGAGACTATTTTGTAGACGATAATGAATTGCCCCCTTTAATAACAAGTGGATTTAATCCGTCTTTTATCGCG
CC151-s483  631 ACAAGAGGACCTAATGCTAGAGACTATTTTGTAGACGATAATGAATTGCCCCCTTTAATAACAAGTGGATTTAATCCGTCTTTTATCGCG
CC151-s909  631 ACAAGAGGACCTAATGCTAGAGACTATTTTGTAGACGATAATGAATTGCCCCCTTTAATAACAAGTGGATTTAATCCGTCTTTTATCGCG
CC479-s15   631 ACAAGAGGACCTAATGCTAGAGACTATTTTGTAGACGATAATGAATTGCCCCCTTTAATAACAAGTGGATTTAATCCGTCTTTTATCGCG
CC479-s28   631 ACAAGAGGACCTAATGCTAGAGACTATTTTGTAGACGATAATGAATTGCCCCCTTTAATAACAAGTGGATTTAATCCGTCTTTTATCGCG
```

```
CC479-s58   631 ACAAGAGGACCTAATGCTAGAGACTATTTTGTAGACGATAATGAATTGCCCCCTTTAATAACAAGTGGATTTAATCCGTCTTTTATCGCG

CC133-s139  721 ACAGTATCTCACGAAAAAGATTCAGGCGATACGAGCGAATTTGAAATTACTTACGGTAGAAATATGGATGTTACCTATGCAACCTACCTT
CC151-s31   721 ACAGTATCTCACGAAAAAGATTCAGGCGATACGAGCGAATTTGAAATTACTTACGGTAGAAATATGGATGTTACCTATGCAACCTACCTT
CC151-s483  721 ACAGTATCTCACGAAAAAGATTCAGGCGATACGAGCGAATTTGAAATTACTTACGGTAGAAATATGGATGTTACCTATGCAACCTACCTT
CC151-s909  721 ACAGTATCTCACGAAAAAGATTCAGGCGATACGAGCGAATTTGAAATTACTTACGGTAGAAATATGGATGTTACCTATGCAACCTACCTT
CC479-s15   721 ACAGTATCTCACGAAAAAGATTCAGGCGATACGAGCGAATTTGAAATTACTTACGGTAGAAATATGGATGTTACCTATGCAACCTACCTT
CC479-s28   721 ACAGTATCTCACGAAAAAGATTCAGGCGATACGAGCGAATTTGAAATTACTTACGGTAGAAATATGGATGTTACCTATGCAACCTACCTT
CC479-s58   721 ACAGTATCTCACGAAAAAGATTCAGGCGATACGAGCGAATTTGAAATTACTTACGGTAGAAATATGGATGTTACCTATGCAACCTACCTT

CC133-s139  811 CCTAAACTTGGTCTTTATCCAGAAAGAAAACATAATGAATTTGTAAACAGAAACTTTGTGGTCAAATATGAAGTGAATTGGAAAACGTAC
CC151-s31   811 CCTAAACAAGGTCTTTATCCAGAAAGAAAACATAATGAATTTGTAAACAGAAACTTTGTGGTCAAATATGAAGTGAATTGGAAAACGCAC
CC151-s483  811 CCTAAACAAGGTCTTTATCCAGAAAGAAAACATAATGAATTTGTAAACAGAAACTTTGTGGTCAAATATGAAGTGAATTGGAAAACGCAC
CC151-s909  811 CCTAAACAAGGTCTTTATCCAGAAAGAAAACATAATGAATTTGTAAACAGAAACTTTGTGGTCAAATATGAAGTGAATTGGAAAACGCAC
CC479-s15   811 CCTAAACTTGGTCTTTATCCAGAAAGAAAACATAATGAATTTGTAAACAGAAACTTTGTGGTCAAATATGAAGTGAATTGGAAAACGTAC
CC479-s28   811 CCTAAACTTGGTCTTTATCCAGAAAGAAAACATAATGAATTTGTAAACAGAAACTTTGTGGTCAAATATGAAGTGAATTGGAAAACGTAC
CC479-s58   811 CCTAAACTTGGTCTTTATCCAGAAAGAAAACATAATGAATTTGTAAACAGAAACTTTGTGGTCAAATATGAAGTGAATTGGAAAACGTAC

CC133-s139  901 GAAATTAAAGTAAAGGGGCACAACTAA
CC151-s31   901 GAAATTAAAGTAAAGGGGCACAACTAA
CC151-s483  901 GAAATTAAAGTAAAGGGGCACAACTAA
CC151-s909  901 GAAATTAAAGTAAAGGGGCACAACTAA
CC479-s15   901 GAAATTAAAGTAAAGGGGCACAACTAA
CC479-s28   901 GAAATTAAAGTAAAGGGGCACAACTAA
CC479-s58   901 GAAATTAAAGTAAAGGGGCACAACTAA
```

Figure S1 (a). Alignment of *lukM* of seven *S. aureus* isolates obtained from cases of bovine mastitis. Sequences obtained from whole genome sequence (WGS) from seven isolates using reference *lukM* sequence (GenBank accession: 1262967). Isolates were selected based on CC and LukMF′ production potential (high LukMF′ production = CC479, low LukMF′ producing = CC151, CC133). Black area indicates consensus region of genes.

```
CC133-s139    1 ATGAAATTTAAGAATATAGTCAAATCATCAGTCGCTACATCAATTACATTAATCATGCTATCAAATACAGTTGATGCAGCTCAACATATC
CC151-s31     1 ATGAAAATTAAGAATATAGTCAAATCATCAGTCGCTACATCAATTACATTTATCATGCTATCAAATACAGTTGATGCAGCTCAACATATC
CC151-s483    1 ATGAAAATTAAGAATATAGTCAAATCATCAGTCGCTACATCAATTACATTTATCATGCTATCAAATACAGTTGATGCAGCTCAACATATC
CC151-s909    1 ATGAAAATTAAGAATATAGTCAAATCATCAGTCGCTACATCAATTACATTTATCATGCTATCAAATACAGTTGATGCAGCTCAACATATC
CC479-s15     1 ATGAAATTTAAGAATATAGTCAAATCATCAGTCGCTACATCAATTACATTAATCATGCTATCAAATACAGTTGATGCAGCTCAACATATC
CC479-s28     1 ATGAAATTTAAGAATATAGTCAAATCATCAGTCGCTACATCAATTACATTAATCATGCTATCAAATACAGTTGATGCAGCTCAACATATC
CC479-s58     1 ATGAAATTTAAGAATATAGTCAAATCATCAGTCGCTACATCAATTACATTAATCATGCTATCAAATACAGTTGATGCAGCTCAACATATC

CC133-s139   91 ACACCTGTCAGCGAGAAAAAAGTGGATGACAAAATCACTTTGTACAAAACGACTGCTACATCAGATTCTGACAAATTAAAAATTTCTCAA
CC151-s31    91 ACACCTGTCAGCGAGAAAAAAGTGGATGACAAAATCACTTTGTACAAAACGACTGCTACATCAGATTCTGACAAATTAAAAATTTCTCAA
CC151-s483   91 ACACCTGTCAGCGAGAAAAAAGTGGATGACAAAATCACTTTGTACAAAACGACTGCTACATCAGATTCTGACAAATTAAAAATTTCTCAA
CC151-s909   91 ACACCTGTCAGCGAGAAAAAAGTGGATGACAAAATCACTTTGTACAAAACGACTGCTACATCAGATTCTGACAAATTAAAAATTTCTCAA
CC479-s15    91 ACACCTGTCAGCGAGAAAAAAGTGGATGACAAAATCACTTTGTACAAAACGACTGCTACATCAGATTCTGACAAATTAAAAATTTCTCAA
CC479-s28    91 ACACCTGTCAGCGAGAAAAAAGTGGATGACAAAATCACTTTGTACAAAACGACTGCTACATCAGATTCTGACAAATTAAAAATTTCTCAA
CC479-s58    91 ACACCTGTCAGCGAGAAAAAAGTGGATGACAAAATCACTTTGTACAAAACGACTGCTACATCAGATTCTGACAAATTAAAAATTTCTCAA

CC133-s139  181 ATTCTAACTTTTAATTTTATTAAAGACAAAAGTTATGATAAAGACACATTAATACTAAAAGCTGCCGGAAACATTTACTCAGGCTATACC
CC151-s31   181 ATTCTAACTTTTAATTTTATTAAAGACAAAAGTTATGATAAAGACACATTAATACTAAAAGCTGCCGGAAACATTTACTCAGGCTATACC
CC151-s483  181 ATTCTAACTTTTAATTTTATTAAAGACAAAAGTTATGATAAAGACACATTAATACTAAAAGCTGCCGGAAACATTTACTCAGGCTATACC
CC151-s909  181 ATTCTAACTTTTAATTTTATTAAAGACAAAAGTTATGATAAAGACACATTAATACTAAAAGCTGCCGGAAACATTTACTCAGGCTATACC
CC479-s15   181 ATTCTAACTTTTAATTTTATTAAAGACAAAAGTTATGATAGAGACACATTAATACTAAAAGCTGCCGGAAACATTTACTCAGGCTATACC
CC479-s28   181 ATTCTAACTTTTAATTTTATTAAAGACAAAAGTTATGATAGAGACACATTAATACTAAAAGCTGCCGGAAACATTTACTCAGGCTATACC
CC479-s58   181 ATTCTAACTTTTAATTTTATTAAAGACAAAAGTTATGATAGAGACACATTAATACTAAAAGCTGCCGGAAACATTTACTCAGGCTATACC

CC133-s139  271 CAACCCACTTCTGATAGTAGTATAAATTCACAATTTTATTGGGGAGCTAAGTATAATGTTTTTGTTAGCTCGGAGTCCAAAGATTCTGTA
CC151-s31   271 CAACCCACTTCTGATAGTAGTATAAATTCACAATTTTATTGGGGAGCTAAGTATAATGTTTTTGTTAGCTCGGAGTCCAAAGATTCTGTA
CC151-s483  271 CAACCCACTTCTGATAGTAGTATAAATTCACAATTTTATTGGGGAGCTAAGTATAATGTTTTTGTTAGCTCGGAGTCCAAAGATTCTGTA
CC151-s909  271 CAACCCACTTCTGATAGTAGTATAAATTCACAATTTTATTGGGGAGCTAAGTATAATGTTTTTGTTAGCTCGGAGTCCAAAGATTCTGTA
CC479-s15   271 CAACCCACTTCTGATAGTAGTATAAATTCACAATTTTATTGGGGAGCTAAGTATAATGTTTTTGTTAGCTCGGAGTCCAAAGATTCTGTA
CC479-s28   271 CAACCCACTTCTGATAGTAGTATAAATTCACAATTTTATTGGGGAGCTAAGTATAATGTTTTTGTTAGCTCGGAGTCCAAAGATTCTGTA
CC479-s58   271 CAACCCACTTCTGATAGTAGTATAAATTCACAATTTTATTGGGGAGCTAAGTATAATGTTTTTGTTAGCTCGGAGTCCAAAGATTCTGTA

CC133-s139  361 AATATTGTTGACTACGCGCCTAAAAATCAAAATGAAGAATTTCAAGTTCAACAAACATTAGGTTATTCATATGGCGGAGATATTAATATA
CC151-s31   361 AATATTGTTGACTACGCGCCTAAAAATCAAAATGAAGAATTTCAAGTTCAACAAACATTAGGTTATTCATATGGCGGAGATATTAATATA
CC151-s483  361 AATATTGTTGACTACGCGCCTAAAAATCAAAATGAAGAATTTCAAGTTCAACAAACATTAGGTTATTCATATGGCGGAGATATTAATATA
CC151-s909  361 AATATTGTTGACTACGCGCCTAAAAATCAAAATGAAGAATTTCAAGTTCAACAAACATTAGGTTATTCATATGGCGGAGATATTAATATA
CC479-s15   361 AATATTGTTGACTACGCGCCTAAAAATCAAAATGAAGAATTTCAAGTTCAACAAACATTAGGTTATTCATATGGCGGAGATATTAATATA
CC479-s28   361 AATATTGTTGACTACGCGCCTAAAAATCAAAATGAAGAATTTCAAGTTCAACAAACATTAGGTTATTCATATGGCGGAGATATTAATATA
CC479-s58   361 AATATTGTTGACTACGCGCCTAAAAATCAAAATGAAGAATTTCAAGTTCAACAAACATTAGGTTATTCATATGGCGGAGATATTAATATA

CC133-s139  451 ATAAATGGATTAACTGGTGGATTGAATGGGTCAAAATCATTTTCAGAAACGATTAATTATAAGCAAGAAAGCTACAGAACTACGATTGAT
CC151-s31   451 ATAAATGGATTAACTGGTGGATTGAACGGGTCAAAATCATTTTCAGAAACGATTAATTATAAGCAAGAAAGCTACAGAACTACGATTGAT
```

```
CC151-s483  451 ATAAATGGATTAACTGGTGGATTGAACGGGTCAAAATCATTTTCAGAAACGATTAATTATAAGCAAGAAAGCTACAGAACTACGATTGAT
CC151-s909  451 ATAAATGGATTAACTGGTGGATTGAACGGGTCAAAATCATTTTCAGAAACGATTAATTATAAGCAAGAAAGCTACAGAACTACGATTGAT
CC479-s15   451 ATAAATGGATTAACTGGTGGATTGAATGGGTCAAAATCATTTTCAGAAACGATTAATTATAAGCAAGAAAGCTACAGAACTACGATTGAT
CC479-s28   451 ATAAATGGATTAACTGGTGGATTGAATGGGTCAAAATCATTTTCAGAAACGATTAATTATAAGCAAGAAAGCTACAGAACTACGATTGAT
CC479-s58   451 ATAAATGGATTAACTGGTGGATTGAATGGGTCAAAATCATTTTCAGAAACGATTAATTATAAGCAAGAAAGCTACAGAACTACGATTGAT

CC133-s139  541 AGGAAAACAAATCATAAATCAATCGGCTGGGGTGTCGAGGCACATAAAATCATGAATAATGGTTGGGGGCCATATGGCAGAGATAGTAGT
CC151-s31   541 AGGAAAATAAATCACAAATTAATCGGCTGGGGTGTCGAGGCACATAAAATCATGAATAATGGTTGGGGACCATATGGCAGAGATAGTAGT
CC151-s483  541 AGGAAAATAAATCACAAATTAATCGGCTGGGGTGTCGAGGCACATAAAATCATGAATAATGGTTGGGGACCATATGGCAGAGATAGTAGT
CC151-s909  541 AGGAAAATAAATCACAAATTAATCGGCTGGGGTGTCGAGGCACATAAAATCATGAATAATGGTTGGGGACCATATGGCAGAGATAGTAGT
CC479-s15   541 AGGAAAACAAATCATAAATCAATCGGCTGGGGTGTCGAGGCACATAAAATCATGAATAATGGTTGGGGACCATATGGCAGAGATAGTAGT
CC479-s28   541 AGGAAAACAAATCATAAATCAATCGGCTGGGGTGTCGAGGCACATAAAATCATGAATAATGGTTGGGGACCATATGGCAGAGATAGTAGT
CC479-s58   541 AGGAAAACAAATCATAAATCAATCGGCTGGGGTGTCGAGGCACATAAAATCATGAATAATGGTTGGGGACCATATGGCAGAGATAGTAGT

CC133-s139  631 GATTCATTATATGGAAACGAACTATTTTTAGGTGGCAGACAGAGTAGCTCGAATGCTAATCAAAATTTCTTACCAACACATCAAATGCCC
CC151-s31   631 GATTCATTATATGGAAACGAACTATTTTTAGGTGGCAGACAGAGTAGCTCGAATGCTAATCAAAATTTCTTACCAACACATCAAATGCCC
CC151-s483  631 GATTCATTATATGGAAACGAACTATTTTTAGGTGGCAGACAGAGTAGCTCGAATGCTAATCAAAATTTCTTACCAACACATCAAATGCCC
CC151-s909  631 GATTCATTATATGGAAACGAACTATTTTTAGGTGGCAGACAGAGTAGCTCGAATGCTAATCAAAATTTCTTACCAACACATCAAATGCCC
CC479-s15   631 GATTCATTATATGGAAACGAACTATTTTTAGGTGGCAGACAGAGTAGCTCGAATGCTAATCAAAATTTCTTACCAACACATCAAATGCCC
CC479-s28   631 GATTCATTATATGGAAACGAACTATTTTTAGGTGGCAGACAGAGTAGCTCGAATGCTAATCAAAATTTCTTACCAACACATCAAATGCCC
CC479-s58   631 GATTCATTATATGGAAACGAACTATTTTTAGGTGGCAGACAGAGTAGCTCGAATGCTAATCAAAATTTCTTACCAACACATCAAATGCCC

CC133-s139  721 ATATTAGCACGTGGTAATTTCAATCCAGAATTTATAAGCGTACTTTCTCACAAACAAAAGGATGTTAAAAAATCTAAAATTAAAGTGACT
CC151-s31   721 ATATTAGCACGTGGTAATTTCAATCCAGAATTTATAAGCGTACTTTCTCACAAACAAAAGGATGTTAAAAAATCTAAAATTAAAGTGACT
CC151-s483  721 ATATTAGCACGTGGTAATTTCAATCCAGAATTTATAAGCGTACTTTCTCACAAACAAAAGGATGTTAAAAAATCTAAAATTAAAGTGACT
CC151-s909  721 ATATTAGCACGTGGTAATTTCAATCCAGAATTTATAAGCGTACTTTCTCACAAACAAAAGGATGTTAAAAAATCTAAAATTAAAGTGACT
CC479-s15   721 ATATTAGCACGTGGTAATTTCAATCCAGAATTTATAAGCGTACTTTCTCACAAACAAAAGGATGTTAAAAAATCTAAAATTAAAGTGACT
CC479-s28   721 ATATTAGCACGTGGTAATTTCAATCCAGAATTTATAAGCGTACTTTCTCACAAACAAAAGGATGTTAAAAAATCTAAAATTAAAGTGACT
CC479-s58   721 ATATTAGCACGTGGTAATTTCAATCCAGAATTTATAAGCGTACTTTCTCACAAACAAAAGGATGTTAAAAAATCTAAAATTAAAGTGACT

CC133-s139  811 TATCAAAGAGAAATGGATCGGTATGAAAATTTTTGGAACAACTTGCACTGGATAGGTTATAATATTAAGAATCAAAAGAGAGCAACACAC
CC151-s31   811 TATCAAAGACAAATGGATCGGTATGAAAATTTTTGGAACAACTTGCACTGGATAGGTTATAATATTAAGAATCAAAAGAGAGCAACACAC
CC151-s483  811 TATCAAAGACAAATGGATCGGTATGAAAATTTTTGGAACAACTTGCACTGGATAGGTTATAATATTAAGAATCAAAAGAGAGCAACACAC
CC151-s909  811 TATCAAAGACAAATGGATCGGTATGAAAATTTTTGGAACAACTTGCACTGGATAGGTTATAATATTAAGAATCAAAAGAGAGCAACACAC
CC479-s15   811 TATCAAAGAGAAATGGATCGGTATGAAAATTTTTGGAACAACTTGCACTGGATAGGTTATAATATTAAGAATCAAAAGAGAGCAACACAC
CC479-s28   811 TATCAAAGAGAAATGGATCGGTATGAAAATTTTTGGAACAACTTGCACTGGATAGGTTATAATATTAAGAATCAAAAGAGAGCAACACAC
CC479-s58   811 TATCAAAGAGAAATGGATCGGTATGAAAATTTTTGGAACAACTTGCACTGGATAGGTTATAATATTAAGAATCAAAAGAGAGCAACACAC

CC133-s139  901 ACATCAATTTATGAAATTGATTGGGAAAAACACACGGTTAAATTAGTAGCTTCGCAATCTAGCGAATAA
CC151-s31   901 ACATCAATTTATGAAATTGATTGGGAAAAACACACGGTTAAATTAGTAGCTTCGCAATCTAGCGAATAA
CC151-s483  901 ACATCAATTTATGAAATTGATTGGGAAAAACACACGGTTAAATTAGTAGCTTCGCAATCTAGCGAATAA
CC151-s909  901 ACATCAATTTATGAAATTGATTGGGAAAAACACACGGTTAAATTAGTAGCTTCGCAATCTAGCGAATAA
CC479-s15   901 ACATCAATTTATGAAATTGATTGGGAAAAACACACGGTTAAATTAGTAGCTTCGCAATCTAGCGAATAA
CC479-s28   901 ACATCAATTTATGAAATTGATTGGGAAAAACACACGGTTAAATTAGTAGCTTCGCAATCTAGCGAATAA
CC479-s58   901 ACATCAATTTATGAAATTGATTGGGAAAAACACACGGTTAAATTAGTAGCTTCGCAATCTAGCGAATAA
```

Figure S1 (**b**). Alignment of *lukF′* of seven *S. aureus* isolates obtained from cases of bovine mastitis. Sequences were obtained from whole genome sequence (WGS) from seven isolates using reference *lukF* sequence (GenBank accession: 1262954)

```
CC133-s139    1 GTTTTTCAGGTATATTTCTATACAACTATAATGAACTTTTTTAATTATTGATCGTGTTTGAATGTTTTTTGGGTGTAACAATACTATTTT
CC151-s31     1 GTTTTTCAGGTATATTTCTATACAACTATAATGAACTTTTTTAATTATTGATCGTGTTTGAATGCTTTTTG-GTGTAAAAATACTATTTT
CC151-s483    1 GTTTTTCAGGTATATTTCTATACAACTATAATGAACTTTTTTAATTATTGATCGTGTTTGAATGCTTTTTG-GTGTAAAAATACTATTTT
CC151-s909    1 GTTTTTCAGGTATATTTCTATACAACTATAATGAACTTTTTTAATTATTGATCGTGTTTGAATGCTTTTTG-GTGTAAAAATACTATTTT
CC479-s15     1 GTTTTTCAGGTATATTTCTATACAACTATAATGAACTTTTTTAATTATTGATCGTGTTTGAATGTTTTTTGGGTGTAACAATACTATTTT
CC479-s28     1 GTTTTTCAGGTATATTTCTATACAACTATAATGAACTTTTTTAATTATTGATCGTGTTTGAATGTTTTTTGGGTGTAACAATACTATTTT
CC479-s58     1 GTTTTTCAGGTATATTTCTATACAACTATAATGAACTTTTTTAATTATTGATCGTGTTTGAATGTTTTTTGGGTGTAACAATACTATTTT

CC133-s139   91 AGAGATTTATTAACCTTATTGAATTTTTAATAAATTTTTAATTATATAATTATGAATTATGTAATAGTATAAATTTGTATTAAAAATATA
CC151-s31    90 AGGGATTCATTAACCTTATTGAATTTTTAATAAATTTTTAATTATATAATTATGAATTATGTAATAGTATAAATTTGTATTAAAAATATA
CC151-s483   90 AGGGATTCATTAACCTTATTGAATTTTTAATAAATTTTTAATTATATAATTATGAATTATGTAATAGTATAAATTTGTATTAAAAATATA
CC151-s909   90 AGGGATTCATTAACCTTATTGAATTTTTAATAAATTTTTAATTATATAATTATGAATTATGTAATAGTATAAATTTGTATTAAAAATATA
CC479-s15    91 AGAGATTTATTAACCTTATTGAATTTTTAATAAATTTTTAATTATATAATTATGAATTATGTAATAGTATAAATTTGTATTAAAAATATA
CC479-s28    91 AGAGATTTATTAACCTTATTGAATTTTTAATAAATTTTTAATTATATAATTATGAATTATGTAATAGTATAAATTTGTATTAAAAATATA
CC479-s58    91 AGAGATTTATTAACCTTATTGAATTTTTAATAAATTTTTAATTATATAATTATGAATTATGTAATAGTATAAATTTGTATTAAAAATATA

CC133-s139  181 AAGAGAAAGAAAGTGAAACT
CC151-s31   180 AAGAGAAAGAAAGTGAAACT
CC151-s483  180 AAGAGAAAGAAAGTGAAACT
CC151-s909  180 AAGAGAAAGAAAGTGAAACT
CC479-s15   181 AAGAGAAAGAAAGTGAAACT
CC479-s28   181 AAGAGAAAGAAAGTGAAACT
CC479-s58   181 AAGAGAAAGAAAGTGAAACT
```

Figure S1 (**c**). Alignment of the putative promotor region (the region of 200 base pairs before the start of *lukM*, containing the

putative Tata box and promoter elements) of *lukM-lukF′* operon of seven *S. aureus* isolates obtained from cases of bovine mastitis

```
CC133-s139-WGS    1 ATGCATAAGTTAGCACATAC
CC151-s31-WGS     1 ATGCATAAGTTAGCACATAC
CC151-s483-WGS    1 ATGCATAAGTTAGCACATAC
CC151-s505-PCR    1 ATGCATAAGTTAGCACATAC
CC151-s605-PCR    1 ATGCATAAGTTAGCACATAC
CC151-s909-WGS    1 ATGCATAAGTTAGCACATAC
CC479-s15-WGS     1 AAGCATAAGTTAGCACATAC
CC479-s28-WGS     1 AAGCATAAGTTAGCACATAC
CC479-s58-WGS     1 AAGCATAAGTTAGCACATAC
CC479-s192-PCR    1 AAGCATAAGTTAGCACATAC
CC479-s245-PCR    1 AAGCATAAGTTAGCACATAC
CC479-s670-PCR    1 AAGCATAAGTTAGCACATAC
```

Figure S1 (**d**). Alignment of the first 20 nucleotides of rot in 12 *S. aureus* isolates obtained from cases of bovine mastitis. Sequences of seven isolates obtained from the genome after whole genome sequencing (labeled WGS), five additional sequences were obtained from sequenced PCR products (labeled PCR).

Table S1. Number of *S. aureus* of different lineages found among bovine isolates, with percentage of *lukM-lukF′* positive *S. aureus* among these lineages

	**Portugal**	**Sweden**[1]	**Germany**[1]	**Japan**	**Switzerland/Germany**	**South Africa**
**Sample** / **Lineage**	Nasal swabs	Clinical mastitis	Subclinical mastitis	Subclinical mastitis	Mastitis	Subclinical mastitis
ST133-CC133		20(100)[2]	21(100)		6 (100)	
ST124-CC133	13 0)					
ST352-CC97[3]	13 (100)			24 (92)		71 (100)
ST97-CC97[4]		13 (85)	9 (0)	16 (6)	23 (0)	63 (0)
ST151-CC151			98 (100)		61 (100)	
ST705-CC705[5]		29 (100)		9 (100)		10 (100)
ST479-CC479		1(100)	33 (97)			
ST30-CC20[6]		1 (0)		14 (0)	9 (11)	
ST5 -CC5				4 (0)	2 (0)	
ST7-CC7[7]	8 (0)		1 (0)	1 (0)		
ST398-CC398			8(0)		2(0)	
ST8-CC8		1 (0)		6 (0)	11 (0)	1 (0)
ST508-CC45		3 (0)	5 (0)	3 (0)		1 (100)
Other CC		2 (0)	14 (0)	33 (0)	14 (0)	
**Total**	**34 (38)**	**70 (90)**	**189 (80)**	**110 (29)**	**128 (53)**	**146 (56)**
**References**	[34]	[32]	[16]	[31]	[33]	[30]

1. Only Clonal Complex (CC) data available in these studies
2. Number of *S. aureus* of this lineage found, with percentage of *lukM-lukF′* positive *S. aureus* among this lineage in brackets
3. Includes single locus variant (SLV) ST1366
4. Includes SLV ST124, ST1367
5. Includes SLV ST351, ST1363, ST1363, ST1365
6. Includes SLV ST1368, ST1370
7. Includes SLV ST789
